# Veterans are not the only ones suffering from posttraumatic stress symptoms: what do we know about dependents’ secondary traumatic stress?

**DOI:** 10.1007/s00127-016-1292-6

**Published:** 2016-10-21

**Authors:** Julia Diehle, Samantha K. Brooks, Neil Greenberg

**Affiliations:** 1 King’s Centre for Military Health Research, Department of Psychological Medicine, Institute of Psychiatry, King’s College London, Weston Education Centre, 10 Cutcombe Road, London, SE5 9RJ UK; 2 Department of Psychological Medicine, King’s College London, London, UK; 3 Academic Department of Military Mental Health, Department of Psychological Medicine, King’s College London, London, UK

**Keywords:** PTSD, STS, Vicarious trauma, Veterans, Family

## Abstract

**Purpose:**

Previous research has mainly focused on veterans’ mental health problems, especially on posttraumatic stress disorder (PTSD). Less is known about the impact that the veteran’s experienced potentially traumatic events (PTEs) might have on their significant others. Therefore, we reviewed the scientific literature to find out what is known about the prevalence of secondary traumatic stress (STS) in significant others of veterans.

**Methods:**

We systematically searched Pubmed, PsycINFO, Embase, Cochrane Library and PILOTS for relevant articles. This search resulted in 3100 records from which we included 48 articles.

**Results:**

Two studies that reported on parental PTSD did not find evidence that parents were affected by their offspring’s experience. Nine studies that reported on PTSD in mainly adult children of veterans found only scant evidence that children were affected by their parent’s experienced PTE. Twenty-seven studies investigated PTSD symptoms in partners of veterans. Here results varied largely between studies with PTSD rates between 0 and 51 %.

**Conclusions:**

Overall, we found the strongest evidence of STS in partners of help-seeking veterans with PTSD. The lack of clarity provided by the currently available evidence suggests a pressing need for further work to examine this subject in more detail.

## Background

Armed forces (AF) personnel are required to deploy to conflict zones which exposes them to potentially traumatic events (PTEs) placing them at risk of developing posttraumatic stress disorder (PTSD). Various studies demonstrate that active military personnel and service leavers (hereafter referred collectively as veterans) often suffer from PTSD and other mental health problems [1]. In particular those deployed in combat roles appear more likely to develop PTSD (see [2] for an overview).

However, as well as impacting on the veteran, the veteran’s PTEs may also impact on significant others who are at risk of developing vicarious trauma or secondary traumatic stress (referred to as STS hereafter). STS may affect people who have close relationships with those who experienced a first-hand trauma. The symptoms of STS mirror those of PTSD and include re-experiencing, avoidance and hyperarousal symptoms in reference to an event which they themselves have not experienced [3, 4].

STS has been reported in mental health professionals treating trauma survivors who have been found to develop trauma symptoms in reference to the traumatic experience of their clients [5]. However, the evidence about whether family members are at risk of developing STS is mixed. A meta-analytic review on second generation Holocaust survivors showed that survivors who presented clinically having experienced a PTE themselves had poorer outcomes than non-Holocaust controls but non-clinical survivors did not differ significantly from non-clinical controls [6]. Another study found that adult children of Holocaust survivors whose mothers had suffered from PTSD were at greater risk to have PTSD themselves, whereas no such association was found with paternal PTSD [7]. Recently, research has focused on the impact that traumatic experiences of deployed veterans might have on family members. In a meta-analytic review, a moderate association was found between the severity of a veteran’s PTSD and psychological distress in their partner [8]. With respect to children, previous research did not find an association between deployment and children’s internalizing symptoms like depression and anxiety, and externalizing symptoms like aggression, or academic problems [9]. However, the presence of mental health problems of the deployed parent was not examined.

Thus, whilst there are suggestions that veterans’ traumatic experiences might also affect significant others, the literature on this subject has not been systematically reviewed to identify if veterans’ significant others suffer from PTSD or STS. Specifically, the objective of the current review was to examine studies that reported PTSD or STS in significant others of veterans and to explore if PTSD in significant others was associated with a veteran’s traumatic experiences.

## Methodology

### Data sources and search terms

The following databases were searched for eligible articles: Pubmed, PsycINFO, Embase, the Cochrane library and PILOTS database. Search terms were [secondary OR intergenerational OR family] AND [PTSD OR posttraumatic OR traumatic symptom* OR combat disorder] AND [military personnel OR veterans OR reservist*]. Comprehensive search details can be found in Table 1. Each database was searched from inception to January 21, 2016.

**Table 1:** Search Strategies

*Pubmed*. Date of search: 21st January 2016 (Vicarious OR secondary OR intergenerational OR familial OR family[MeSH Terms] OR family[All Fields] OR spouses[MeSH Terms] OR spouse*[All Fields] OR partner[All Fields] OR husband[All Fields] OR wife[All Fields] OR parent[All Fields] OR parents[All Fields] OR mother[All Fields] OR father[All Fields] OR child[All Fields] OR children[All Fields] OR son[All Fields] OR sons[All Fields] OR daughter*[All Fields] OR stepchild[All Fields] OR stepchildren[All Fields] OR brother[All Fields] OR sister[All Fields] OR sibling*[All Fields] OR grandparent*[All Fields] OR grandfather[All Fields] OR grandmother[All Fields] OR grandchild[All Fields] OR grandchildren[All Fields] OR grandson[All Fields] OR granddaughter[All Fields]) AND (Stress Disorders, Traumatic [MeSH Terms] OR ptss[All Fields] OR ptsd[All Fields] OR posttraumatic[All Fields] OR post traumatic[All Fields] OR posttraumatic stress disorder OR post traumatic stress disorder OR traumatic symptom*[All Fields] Or Combat Disorders) AND (veterans[MeSH Terms] OR veterans health[MeSH terms] OR veteran*[All Fields] OR military personnel[MeSH Terms] OR armed forces personnel[All Fields] OR submariner*[All Fields] OR Navy personnel[All Fields] OR Air Force Personnel[All Fields] OR marines[All Fields] OR soldier[All Fields] OR serviceman[All Fields] OR servicemen[All Fields] OR military[All Fields] OR army personnel[All Fields] OR reservist*[All Fields]) No additional limits applied
*Psychinfo* 1806 to 21st January 2016 and *EMBASE* 1980 to 21st January 2016 1. (ptsd or ptss or sts or stsd or posttraumatic or posttraumatic or posttraumatic stress or post traumatic stress or posttraumatic stress symptom? or post traumatic stress symptom? or traumatic symptom?).mp. 2. Exp posttraumatic stress disorder/ 3. Emotional trauma.mp. or exp Emotional Trauma/ 4. (Vicarious or secondary or familial).mp 5. Exp Vicarious Experiences/ 6. Family.mp. or exp Family/ 7. Exp Intergenerational Relations/or intergenerational.mp 8. (Spouse or spouses or partner or husband or wife or parent or parents or mother or father or child or children or stepchild or stepchildren or son or sons or daughter? or brother? or sister? or sibling? or dependent or grandfather or grandmother or grandchild or grandchildren or grandson or granddaughter).ab,sh,id,tw 9. (Military personnel or armed forces personnel or submariner? or Navy personnel or Air Force Personnel or marines or soldier? or serviceman or servicemen or military or army personnel or reservist?).mp. 10. Exp military personnel/ 11. Military reserves.mp. or exp Military Duty Status/ 12. Exp Military Veterans/ 13. Veteran.ab,id,ti,sh. 14. 1 or 2 or 3 15. 4 or 5 or 6 or 7 or 8 16. 9 or 10 or 11 or 12 or 13 17. 14 and 15 and 16 No additional limits applied
*The Cochrane Library* (Wiley Interscience). Date of search: 21st January 2016 #1 (Vicarious or secondary or intergenerational or familial or family or family or spouses or spouse* or partner or husband or wife or parent or parents or mother or father or child or children or son or sons or daughter* or stepchild or stepchildren or brother or sister or sibling* or grandparent* or grandfather or grandmother or grandchild or grandchildren or grandson or granddaughter):ti,ab,kw (Word variations have been searched) #2 (ptsd or ptss or sts or stsd or posttraumatic or post traumatic or posttraumatic stress or post traumatic stress or posttraumatic stress symptom? or post traumatic stress symptom? or traumatic symptom?) #3 (Military personnel or armed forces personnel or submariner? or Navy personnel or Air Force Personnel or marines or soldier? or serviceman or servicemen or military or army personnel or reservist? or Military Personnel or military reserves or Military Veterans or veteran) No additional limits applied.
*PILOTS*—Published International Literature on Traumatic Stress (CSA). Date of search: 21st January 2016 (SU.EXACT(“PTSD (ICD-11)”) OR SU.EXACT(“PTSD (ICD-10)”) OR SU.EXACT(“PTSD (DSM-III)”) OR SU.EXACT(“PTSD (DSM-III-R)”) OR SU.EXACT(“Complex PTSD”) OR SU.EXACT(“PTSD (DSM-IV)”) OR SU.EXACT(“PTSD (DSM-5)”) OR SU.EXACT(“PTSD (ICD-9)”)) AND (SU.EXACT(“Family Members”) or SU.EXACT.EXPLODE(“Intergenerational Effects” OR “Trauma Contagion”)) AND (SU.EXACT(“Military Police Personnel”) OR SU.EXACT(“Military Personnel”)) AND (SU.EXACT(“Family Members”) OR SU.EXACT.EXPLODE(“Intergenerational Effects” OR “Trauma Contagion” OR “vicarious” OR “secondary” OR “intergenerational” OR “familial” OR “family” OR “family” OR “spouses” OR “spouse*” OR “partner” OR “husband” OR “wife” OR “parent” OR “parents” OR “mother” OR “father” OR “child” OR “children” OR “son” OR “sons” OR “daughter*” OR “stepchild” OR “stepchildren” OR “brother” OR “sister” OR “sibling*” OR “grandparent*” OR “grandfather” OR “grandmother” OR “grandchild” OR “grandchildren” OR “grandson” OR “granddaughter”)) AND (SU.EXACT(“Military Police Personnel”) OR SU.EXACT(“Military Personnel” OR “military personnel” OR “armed forces personnel” OR “submariner?” OR “Navy personnel” OR “Air Force Personnel” OR “marines” OR “soldier?” OR “serviceman” OR “servicemen” OR “military” OR “army personnel” OR “reservist?” OR “Military Personnel” OR “military reserves” OR “Military Veterans” OR “veteran*”) No additional limits applied

### In- and exclusion criteria

Studies were included if (a) they were published in peer-reviewed journals; (b) participants were significant others of military personnel (e.g., committed partners; children; parents); and (c) PTSD symptoms (PTSS) were assessed by means of a validated instrument or by clinician assessment. Studies were excluded if one of the following criteria applied: (a) articles presenting case studies; (b) articles that primarily focused on intimate partner violence (IPV) and (c) articles published in languages other than English, Dutch or German. Studies primarily focusing on IPV were excluded since the focus of this review was on STS rather than on PTSD resulting from other stressors.

### Article selection and extraction of information

The first two authors (JD, SB) reviewed all titles and abstracts of retrieved articles independently of each other. Those judged eligible were assessed in full text form and again assessed independently for eligibility. Consensus was generally high and disagreements were discussed to reach one final decision. Since discussions led to agreements in all cases there was no need to consult a third party. From the final list of articles, the following information was extracted into Microsoft Excel spreadsheets: number of military personnel and significant others; sample demographics; study design; deployment/area of duty of military personnel; assessment tools; outcomes on PTSD/STS measures.

## Results

**Fig. 1:**
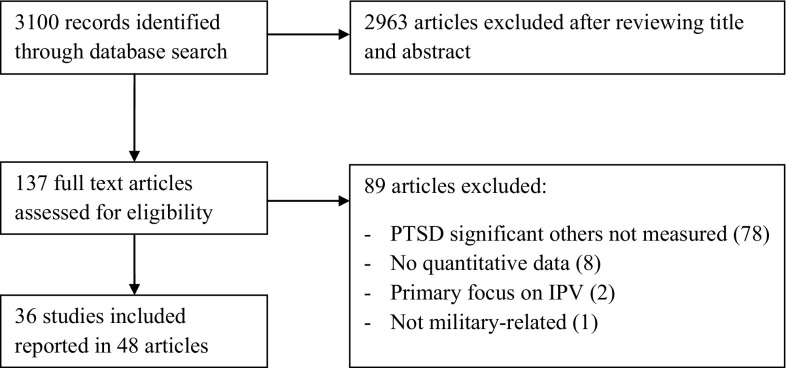
Flowchart diagram

3100 articles were identified through the literature search. The final selection consisted of 36 studies (see Fig. 1, flowchart diagram). Twenty-seven included partners of veterans, two (also) included parents of veterans and nine (also) included children of veterans (see Table 2).

**Table 2:** Description of studies and results

Study [ID]	*N* (couples or dependents of…)	Dependent outcome [% PTSD, mean (SD), correlation with veteran PTSD]	Military-related	Veterans PTSD, %
Partners				
Ahmadi et al. [11]	100	M-PTSD: 51 % >130; *r* = 0.371**	Yes	PCL-M: 30 %
Al-Turkeit et al. [30]	176	PCL: 28.4 % (POW: 39.5 %, IB: 39.1 %; AIA: 15.9 %, retired: 8 %)	Unclear; 81.8 % in Kuwait during invasion	CAPS: 28.9 %
Bjornestad et al. [31]	227	PCL-M: 2.6 %; *r* = 0.217**	Yes	PCL-M: 6.2 %
Blow et al. [12]	661	PCL-C: *r* = 0.13**	Unclear	NA
Bramsen et al. [32]	346 veterans; 161 war victims; 555 controls	IES: controls <war victims*; veterans without PTSD <war victims*	Unclear	76 veterans with PTSD
Caska et al. [33]	130	PCL-C : *r* ^1^ = 0.21*	Yes	PCL-M: 9.4 %
Caspi et al. [24]	129	SPTSS: *r* = 0.25**	Unclear; 67.4 % PTE	SCID: 20 %; 14.5 % war trauma
Church et al. [34]	109	PCL-C: 29.4 %	Unclear	PCL-M: 82.6 %
Collinge et al. [35]	41	PCL-C: 31.8 (11.1)	Unclear	PCL-C: 34.7 (13.6)
Dekel et al. [13]	300	PTSDI: 10 %; *r* = 0.49**	Yes	PTSDI: 27 %
T1: Dekel et al. [36, 37]; Ein-Dor et al. [26]; Solomon et al. [38]	T1: 82/85 ex-POW, 72 combat controls	T1 PTSDI: Ex-POW: 14.1 %; combat controls: 0 %; intrusions: *r* = 0.50**, avoidance: *r* = 0.34**, hyperarousal: *r* = 0.33**; difference in mean between combat controls (lowest), ex-POWs no PTSD and ex-POW with PTSD (highest) **	Yes	T1: 18 ex-POW PTSD; Ex-POW: PTSD-I: 24.8 %; combat controls: PTSD-I: 3.8 %
T2: Greene et al. [39]; Zerach et al. [40, 41]	T2: 148; 66 ex-POW with PTSD; 36 POW no PTSD; 46 combat controls	T2 PTSDI: ex-POW >combat controls*	Yes	T2: 66 PTSD
Dirkzwager et al. [16]	696	SRIP: peacekeepers fulfilling 0 criteria lower mean than peacekeepers fulfilling 1, 2 and 3 criteria**	Unclear	SRIP: 4 % 3 criteria
Erbes et al. [42]	111 deployed, 105 not deployed	PCL-C: 2.4 %, *r* = 0.124*; deployed: 3 %; not deployed: 2 %	Unclear	PCL: All: 2.8 %; deployed: 5 %; not deployed: 1 %
Franciskovic et al. [43]	56	Indirect traumatization questionnaire: 39.3 %	Yes	All PTSD
Gallagher et al. [23]	50	PCL-C: 28 % met criteria for PTSD	Unclear; all PTE	PCL-M: 52 %
Glenn et al. [19]	31	M-PTSD civilian version: 84.5 (20.2)	Unclear	All PTSD
Gorman et al. [20]	212	Short Screening Scale for DSM-IV PTSD: 17 %	Unclear	PCL-M: 11 %
Hamilton et al. [44]	45	PPTSD-R: 34.77 (16.98); TSC-40: 79.00 (27.85)	No	NA
Herzog et al. [45]	54	STS: 14.8 % >44	Probably	PCL-M: 10.8 %
Klaric et al. [46–48]	231	HTQ: veterans with PTSD: 40.3 %; veterans without PTSD: 6.5 %; veterans with PTSD >veterans without PTSD**	Unclear; wives of veterans with PTSD more PTE**	154 with PTSD
Koic et al. [10]	80	M-PTSD: 30 % veterans with PTSD; 0 % veterans without PTSD	Yes	40 with PTSD
Lester et al. [49]	163	PDS >16: enlisted: 14.2 %; officers: 6 %	Unclear	NA
Lester et al. [50]	2073	PCL >30: 31 %	Unclear	NA
Melvin et al. [21]	47 non-deployed	PCL (>29): 34 %; 2 % in absence of own PTE	Some	NA
Miller et al. [28]; Wolf et al. [51, 52]	287	CAPS: 14.3 % PTSD	Some: 6.5 % combat-related trauma	CAPS: 42.9 %
O’Toole et al. [29]	240	CIDI lifetime diagnosis: 18.6 %	Unclear	CAPS lifetime combat-related PTSD: 24.7 %; CIDI lifetime diagnosis: 8.7 %
Renshaw et al. [22]	190	PCL >44: 30.5 %; 56.1 % of these unrelated to veteran’s military experience; 15.5 % solely due to veteran’s military experience	Some	PCL: all >34
Parents				
Caspi et al. [15]	67	SPTSS: 2.71; 1.98 below cut-off of 4	Unclear, 76.1 % PTE	SCID: 9 %
Dirkzwager et al. [16]	329	SRIP: no significant difference between parents of veterans with 0 criteria, parents of veterans with 1 criterion; parents of veterans with 2 criteria and parents of veterans with 3 criteria	Unclear	SRIP: 27 with 3 criteria
Children				
Barnes et al. [53]	121	PCL: children of OIF parent >children of non-deployed and civilian parents*; children of OIF parents with European American ethnicity >children OIF parents with non-European American ethnicity*	Unclear	NA
Beckham et al. [18]	40	MMPI PK: 45 % >18	Unclear	All PTSD
Davidson et al. [14]	50 veterans; 33 civilians	M-PTSD: no significant differences between veterans with PTSD, veterans without PTSD and civilians; veterans with PTSD *r* = −0.02; veterans no PTSD *r* = −0.23; civilian fathers: *r* = 0.23 all rs >0.05	Unclear	NA
Dishtein et al. [54]	92	IES: veterans with PTSD >veterans no PTSD*	Unclear	46 PTSD
Glenn et al. [19]	29	M-PTSD civilian version: 75.6 (21.8)	Unclear	All PTSD
Gorman et al. [55]	642,397	ICD-9 diagnosis 1.2 %	Unclear	NA
Motta et al. [56]	45 veterans; 47 civilians	IES and MMPI-2: no significant difference between children of veterans and children of civilians; both group means not in clinical range	Unclear	NA
Suozzi et al. [57]	53 children of 40 veterans	IES- intrusion and IES avoidance: no significant difference between high combat exposure veterans and low combat exposure veterans; means for both groups lower than mean in normative sample; MMPI-2 PTSD PK Scale: high combat exposure veterans >low combat exposure veterans*; mean scores not in the clinical range	unclear	high combat exposure veterans: M-PTSD 55.5 % >107
Zerach [58]; Zerach and Aloni [59]; Zerach et al. [60]	44 ex-POW with PTSD, 31 ex-POW without PTSD, 39 combat controls; 98 ex-POW, 90 combat controls	PTSD-I: ex-POW’s with PTSD more symptoms than ex-POW without PTSD and combat controls**; ex-POW more PTSD symptoms than children of combat controls**; mean number of symptoms not in clinical range	Yes	44 ex-POW with PTSD

*M-PTSD* Mississippi Scale for Combat-Related PTSD, *PCL-M* PTSD Checklist-Military Version, *POW* Prisoner(s) of War, *IB* in battle, *AIA* active in army, *CAPS* Clinician Administered PTSD Scale, *PCL-C* PTSD Checklist-Civilian Version, *NA* not available, *IES* Impact of Events Scale, *SPTSS* screen for posttraumatic stress symptoms, *PTE* potential traumatic event, *SCID* Structured Clinical Interview for DSM Disorders, *PTSDI* PTSD inventory, *PPTSD-R* Purdue PTSD Sale-Revised, *TSC* trauma symptom checklist, *HTQ* Harvard trauma questionnaire, *PDS* Posttraumatic Stress Diagnostic Scale, *CIDI* Composite International Diagnostic Interview, *SRIP* self-rating inventory for PTSD, *EA* European American, *OIF* operation Iraqi freedom, *MMPI PK* Minnesota Multiphasic Personality Inventory Keane PTSD Scale, *probably* not stated but deductable from descriptions, *unclear* not stated in relation to what traumatic event, *yes* clearly stated; For PCL criterion >50 if not otherwise stated

* *p* < 0.05; ** *p* < 0.01

^1^Correlation of spouses weighted distress (composite score of PTSD, anx and dep) with partner PTSD

### Partners

In nine studies, potential PTSD of partners was assessed in relation to events that their military partner had experienced. Prevalence of PTSD varied from 0 % [10] to 51 % [11]. Correlations between partners’ PTSD and veterans’ PTSD were reported in eight of the 27 studies. These varied between 0.13 [12] and 0.49 [13].

### Children and parents

In nine studies, potential PTSD in children of veterans was assessed. The majority of studies included adult children only or adult children and adolescents while only two studies included adolescents only or younger children only. Most studies found that although children of veterans scored significantly higher on instruments measuring PTSS than children of civilians, these scores were not in the clinical range. The one study that reported correlations between veterans’ PTSS and children’s PTSS found non-significant results [14].

There were two studies [15, 16] which reported on PTSS in parents of veterans. Neither of them indicated that parents showed elevated symptoms.

## Discussion

Military personnel are often exposed to PTEs which puts them and their significant others at risk to develop trauma-related mental health problems. Therefore, this study examined the presence of trauma-related mental health problems in parents, children and partners of military veterans.

The two studies [15, 16] that reported on PTSS in parents did not find indications for elevated parental traumatic stress reactions. Given that in both studies the proportion of veterans suffering from PTSD were similar (8 and 9 %) but demographics and deployment characteristics and number of participants differed, this indicates that independently of demographics and deployment characteristics and methodology overall, there does not seem to be reason to believe that parents develop STS due to their offspring’s traumatic experience.

The nine studies that investigated probable PTSD in children of veterans yielded inconclusive results. Most studies found that although children of veterans scored on average significantly higher on questionnaires measuring PTSS than children of civilians, these scores were not in the clinical range. Eight of the nine studies did not report in reference to what traumatic event PTSS were measured. Only one study [17] investigated STS symptoms in children of Israeli veterans. In this study, children of ex-POWs reported on average one STS symptom more (three versus two) in relation to veterans’ traumatic events than children of combat exposed veterans. Since the average number of symptoms was, however, low for both groups it can be assumed that only few children endorsed symptoms in the clinical range. Of the eight studies for which it was unclear in relation to what traumatic experience children reported PTSS, only one study found that 45 % of the 40 children of Vietnam veterans who met diagnostic criteria for PTSD scored in the clinical range [18]. However, in a comparable study in which PTSD was investigated in 29 children of Vietnam veterans with a diagnosis of current PTSD it was found that on average, children scored low on the Mississippi Scale for Combat-Related PTSD indicating that only few or none of the children scored in the clinical range [19]. In comparison to studies examining partners’ PTSS, sample sizes of the child studies were smaller, with the exception of the study by Gorman et al. [20]. The variation in the absence or presence of PTSS in children could therefore depend on a lack of power of these studies. For now, however, there is scant evidence that children of military personnel might develop STS in relation to their military parent’s traumatic experience.

Although the majority of studies that met the inclusion criteria for this review examined traumatic stress in partners of military personnel, only few of them focused on STS and assessed whether PTSS were indeed related to their veteran partner’s traumatic experience. Even some of the studies that claimed to investigate secondary trauma in partners did not take into account partners’ primary traumatisation. Studies that were executed in countries in which war or military conflicts took place, for example, showed relatively large proportions of PTSD caseness (up to 51 %, see Table 2) but these studies did not take into account that partners might have been exposed to traumatic events themselves which could have influenced the results. First, partners might have suffered from PTSD due to primary traumatic exposure, and second even if they did not suffer from clinically relevant PTSD the prior exposure to a traumatic event could make them more vulnerable to develop STS. This vulnerability has formerly been highlighted in health care providers [5]. A meta-analytic review identified primary trauma exposure in health care providers treating trauma victims as a risk factor to develop STS.

Two studies that took into account partners’ primary trauma exposure, either by asking about and controlling for own trauma history [21] or by asking about the attribution of symptoms to own or their partners’ trauma history [22], found only small percentages of STS; 2 % (of 34 % PTSD caseness in total) and 4.5 % (of 30.5 % PTSD caseness in total), respectively. These results indicate that rather than being (solely) affected by their military partner’s traumatic experience the majority of partners develops PTSD due to exposure to a PTE independently of their partner. Most of the reviewed studies did not report if partners experienced a PTE themselves, however, Gallagher and colleagues [23] reported that all partners who met criteria for potential PTSD reported a PTE, and Caspi and colleagues [24] also reported that at least 67.4 % of the participating wives had experienced a PTE. Results from the US national comorbidity survey also showed that the lifetime prevalence of trauma exposure in women was 51.2 % [25]. It is thus likely that partners, who were in the large majority women, experienced a traumatic event themselves and that problems were not (only) related to veterans’ trauma exposure.

However, it is highly relevant that studies in which partners of veterans with PTSD were compared to partners of veterans without PTSD, the former reported substantially larger proportions of PTSD and/or higher scores on PTSD measures than partners of veterans without PTSD (see Table 2). Also noteworthy is that in six out of eight studies in which partners of help-seeking veterans participated more than 30 % reported probable PTSD while in the seven studies that included partners of non-help-seeking veterans less than 30 % of the partners reported probable PTSD. These data point towards there being a valid association between veteran’s and partner’s PTSD, especially so for veterans seeking help for PTSD. An explanation for this could be that veterans seeking help suffer less from avoidance symptoms and are more inclined to share their traumatic experience with a therapist and their partner. Veterans who do not seek help might experience more avoidance symptoms, and therefore do not share their experience with their partner which in turn might lead to less secondary traumatic stress in partners. However, studies that investigated correlations between veterans’ and partners’ PTSD overall report that the scale of the association between both outcomes was only modest. Furthermore, Ein-Dor et al. [26] investigated correlations between veterans’ avoidance symptoms and partners’ PTSD symptoms and found that the strength of this correlation did not differ from correlations of veterans’ hyperarousal symptoms and wives’ PTSD symptoms.

Although most partner studies were conducted in large samples, sample sizes varied strongly across studies. However, this did not seem to influence the outcome with respect to partners’ reports of PTSS and STS. In terms of sampling it should be mentioned that only three studies described their sampling method as random sampling. Other studies used convenience and self selection samples. Sampling approach did not, however, influence partners’ reports on PTSS or STSS either.

### Strengths and limitations

The major strengths of the current review are the comprehensive literature search and the broad focus on dependents. However, whilst databases were thoroughly searched, references of retrieved articles were not checked for potentially relevant articles and important authors in the field were not contacted to receive information about potentially relevant papers. Neither were specific journals hand searched for potentially relevant articles.

Due to broad inclusion criteria, the selected studies included various populations differing in demographics, deployment sites, clinical status, etc., and used various measures to assess PTSD and STS (see Tables 2, 3). Therefore, we were only able to present study findings descriptively rather than combining the data in a quantitative synthesis. Although IPV in veterans has been discussed in length elsewhere (see [27]), it is possible that the exclusion of studies focusing primarily on IPV could have led to missing out on studies that could have contributed to more insights in PTSD in significant others of veterans. Another limitation is the exclusive inclusion of studies that had been published in peer-reviewed journals, which might have led to a potential bias in the results.

**Table 3:** Study and sample characteristics

	Study ID
Region the study took place	
USA	[12]; [18]; [19]; [20]; [21]; [22]; [23]; [28, 51, 52]; [31]; [33]; [34]; [35]; [42]; [44]; [45]; [49]; [50]; [53]; [55]; [56]; [57]
Middle East	[11]; [13]; [15]; [17; 58–60]; [24]; [30]; [26; 36–41]; [54]
Europe	[10]; [16]; [32]; [43]; [46–48]
Australia	[14]; [29]
Sample recruitment	
Clinical/help-seeking	[10]; [11]; [13] (partially); [18]; [19]; [21] (partially); [22]; [28, 51, 52]; [33]; [34]; [35]; [43]; [44]; [46–48]; [50]; [54]
Non-clinical	[12]; [14]; [15]; [16]; [17; 58–60]; [20]; [23]; [24]; [26; 36–41]; [29]; [30]; [31]; [32]; [42]; [45]; [49]; [53]; [55]; [56]; [57]
Deployment	
Afghanistan/Iraq	[12]; [20]; [21]; [31]; [33]; [35]; [42]; [45]; [53]
Vietnam	[14]; [18]; [19]; [29]; [44]; [56]; [57]
Other/various sites	[10]; [11]; [13]; [15]; [16]; [17; 58–60]; [22]; [23]; [24]; [26; 36–41]; [28, 51, 52]; [30]; [32]; [34]; [43]; [46–48]; [49]; [50]; [54]; [55]

Several limitations of the selected studies themselves have already been discussed including the assessment of STS in significant others without reports on primary PTE. Another assessment issue concerns the use of questionnaires instead of clinical interviews. Only two studies [28, 29] investigated PTSD in partners by means of (semi-)structured clinical interviews. Both studies found prevalence lower than 20 % although one of them [28] was conducted in a clinical sample. This indicates that questionnaire over-estimate the prevalence of PTSD in partners of veterans.

A big gap in the literature was evident in that there has been minimal research on significant others who are not partners. There were only two studies dedicated to parents and the few child studies mostly focussed on adult offspring of veterans rather than on children and adolescents. Thus, there is a need for more investigation of these populations. Independent of population, however, all future studies on the subject of STS should be concerned about primary traumatisation as well and how this might influence the development of STS.

## Conclusion

In conclusion, the studies of the prevalence of PTSD and STS in significant others of veterans were highly heterogeneous in nature. Overall, there did not appear to be any compelling evidence that parents of military veterans suffered with STS and whilst some studies of veteran’s children suggested they were at increased risk of reporting STS symptoms, these did not appear to be at a clinically significant level. The most compelling evidence was in support of veteran’s partners as being at risk of suffering from STS although the association appeared to be only modest. The strongest evidence of STS was found in partners of help-seeking veterans with PTSD. Given the importance of this topic and the lack of clarity afforded by the currently available evidence suggests a pressing need for further work being required to examine this subject in more detail.
